# Editorial: Women in surgical oncology: 2021

**DOI:** 10.3389/fonc.2022.986189

**Published:** 2022-08-31

**Authors:** Lidia Castagneto-Gissey, Alba Di Leone

**Affiliations:** ^1^ Department of Surgical Sciences, Sapienza University of Rome, Rome, Italy; ^2^ Department of Women’s Health, Children’s Health and Public Health, Agostino Gemelli University Polyclinic [Istituto di Ricovero e Cura a Carattere Scientifico (IRCCS)], Rome, Italy

**Keywords:** surgical oncology, women in surgery, breast, colorectal, ovarian, gastric cancer

Currently, female researchers represent merely a minority, accounting for an estimated 29.3% who end up covering this position worldwide, with a great variability according to each country (1). Specifically, Central Asia exhibits the greatest proportion of female researchers with an estimated 48.2% as opposed to South and West Asia with the lowest count globally (i.e. 18.5%) (1).

In response to such a large gender gap in the scientific research community, the UNESCO Institute for Statistics (UIS) is in the midst of developing new indicators in order to better comprehend the reasons behind women’s decisions to pursue one career over another. Several could be the reasons implicated in limiting and discouraging women’s access to the scientific community, including ancient biases and gender stereotypes. By further understanding such issues, the UIS project concurrently aims at reducing the gender inequality in science, technology, engineering and mathematics (STEM) fields, by possibly promoting reforms in policies and implementing changes in favor of gender equality in all countries with the ultimate goal of empowering women (2).

The present Research Topic spans through various fields of surgical oncology, including breast, ovarian, colorectal, gastric, and esophageal cancer and includes research papers by women involved in oncological surgery.

Breast cancer represents, at present, the most common malignancy among women of all ages. Li et al. focused on retrospectively analyzing the outcomes of three surgical options on disease-free and overall survival rates of young women with breast cancer below the age of 35 years. They found that both survival rates were significantly improved in those patients who underwent breast-conserving surgery compared to mastectomy. On the other hand, Wang et al. used the Surveillance, Epidemiology, and End-results (SEER) database of the US National Cancer Institute registry for the determination of differences in patient survival of each different treatment modality for invasive micropapillary carcinoma (IMPC) of the breast. Authors show how in women with early-stage IMPC, breast-conserving therapy is equivalent to mastectomy in terms of survival outcomes. Xu et al. also assessed breast cancer from a molecular point of view and found that Ferroptosis-related prognostic signature could be proposed as novel biomarkers for the prediction of breast cancer prognosis as they seem connected to the immune microenvironment. Molecular and cellular mechanisms of pyroptosis in patients with breast cancer were looked into also by Ren et al.. The inflammation-dependent programmed cell death mediated by inflammasomes, known as pyroptosis, plays a substantial role in the progression of breast cancer and authors suggest how pyroptosis-related genes could be used as new prognostic biomarkers or even targets for breast cancer treatment.

Epithelial ovarian cancer is one of the most aggressive gynecologic cancers. Gao et al. retrospectively evaluated the prognostic impact of retroperitoneal lymphadenectomy in patients with ovarian clear cell cancer. Authors found no survival benefit in patients undergoing retroperitoneal lymphadenectomy and was not an independent predictor of tumor recurrence. In their study, Jiang et al. introduce a new, minimally invasive surgical approach for the treatment of giant ovarian cysts > 20 cm in diameter. All patients successfully underwent single-port laparoscopic surgery for the removal of serous or mucinous cystadenomas.

Wong et al. determined predictors of morbidity and mortality after palliative surgery in patients with peritoneal carcinomatosis due to various primary malignancies. Authors found elevated preoperative albumin levels and a good Eastern Cooperative Oncology Group (ECOG) performance status were independently associated with better short term outcomes following palliative gastrointestinal surgery, supplying a simplified model to predict superior responders to surgical treatment.

Colorectal cancer is another one of the most common cancers, ranking third as the leading cause of death in both men and women. Hashimoto et al. analyzed immunohistochemical data in order to identify protein expression patterns in stages II-III colorectal cancer that could somehow predict patient outcomes. A high expression of Tenascin-C was identified as a single prognostic marker and was correlated with a worst prognosis in both stages of colorectal cancer.

Although radical resection for gastric cancer is currently the only curative treatment option, recurrence after surgery is most commonly peritoneal. Xiang et al. aimed at establishing a reference value for the creation of treatment strategies by identifying current methods for predicting and preventing peritoneal recurrence following surgical resection. Authors highlight how an early gastric cancer diagnosis and a limited loco-regional extension of the primary tumor reduce the risk of peritoneal recurrence, together with intraperitoneal chemotherapy and adjuvant chemotherapy.

Finally, this Research Topic also includes a Study Protocol of a prospective multicenter study on patient participation for clinical trials (Weis et al.). This study aims to develop patient-centered trial information material for this randomized controlled trial and to increase patient acceptance and compliance with randomized treatment strategies and trials.

Promoting gender equality, dismantling stereotypes, and encouraging women to pursue STEM jobs are all necessary to shift entrenched beliefs. Therefore, Frontiers in Oncology is pleased to provide this Research Topic to highlight the contributions of female researchers in all areas of oncology. The work provided here demonstrates the range of oncology research across the board and shows new developments in theory, experimentation, and methodology with applications to interesting and current issues.

## Author contributions

All authors contributed to manuscript revision, read, and approved the submitted version.

## Conflict of interest

The authors declare that the research was conducted in the absence of any commercial or financial relationships that could be construed as a potential conflict of interest.

## Publisher’s note

All claims expressed in this article are solely those of the authors and do not necessarily represent those of their affiliated organizations, or those of the publisher, the editors and the reviewers. Any product that may be evaluated in this article, or claim that may be made by its manufacturer, is not guaranteed or endorsed by the publisher.
